# Reverse active learning based atrous DenseNet for pathological image classification

**DOI:** 10.1186/s12859-019-2979-y

**Published:** 2019-08-28

**Authors:** Yuexiang Li, Xinpeng Xie, Linlin Shen, Shaoxiong Liu

**Affiliations:** 1 Computer Vision Institute, College of Computer Science and Software Engineering, Shenzhen University, Shenzhen, China; 2 Youtu Lab, Tencent, Shenzhen, China; 3 Marshall Laboratory of Biomedical Engineering, School of Biomedical Engineering, Shenzhen University, Shenzhen, China; 4 Guangdong Key Laboratory of IntelligentInformation Processing, Shenzhen University, Shenzhen, China; 5 The National Engineering Laboratory for Big Data System Computing Technology, Shenzhen University, Shenzhen, China; 6 The Sixth People’s Hospital of Shenzhen, Shenzhen, China

**Keywords:** Pathological image classification, Active learning, Atrous convolution, deep learning

## Abstract

**Background:**

Due to the recent advances in deep learning, this model attracted researchers who have applied it to medical image analysis. However, pathological image analysis based on deep learning networks faces a number of challenges, such as the high resolution (gigapixel) of pathological images and the lack of annotation capabilities. To address these challenges, we propose a training strategy called deep-reverse active learning (DRAL) and atrous DenseNet (ADN) for pathological image classification. The proposed DRAL can improve the classification accuracy of widely used deep learning networks such as VGG-16 and ResNet by removing mislabeled patches in the training set. As the size of a cancer area varies widely in pathological images, the proposed ADN integrates the atrous convolutions with the dense block for multiscale feature extraction.

**Results:**

The proposed DRAL and ADN are evaluated using the following three pathological datasets: BACH, CCG, and UCSB. The experiment results demonstrate the excellent performance of the proposed DRAL + ADN framework, achieving patch-level average classification accuracies (ACA) of 94.10%, 92.05% and 97.63% on the BACH, CCG, and UCSB validation sets, respectively.

**Conclusions:**

The DRAL + ADN framework is a potential candidate for boosting the performance of deep learning models for partially mislabeled training datasets.

## Background

The convolutional neural network (CNN) has been attractive to the community since the AlexNet [1] won the ILSVRC 2012 competition. CNN has become one of the most popular classifiers today in the area of computer vision. Due to outstanding performance of CNN, several researchers start to use it for diagnostic systems. For example, Google Brain [2] proposed a multiscale CNN model for breast cancer metastasis detection in lymph nodes. However, the following challenges arise when employing the CNN for pathological image classification.

**Fig. 1:**
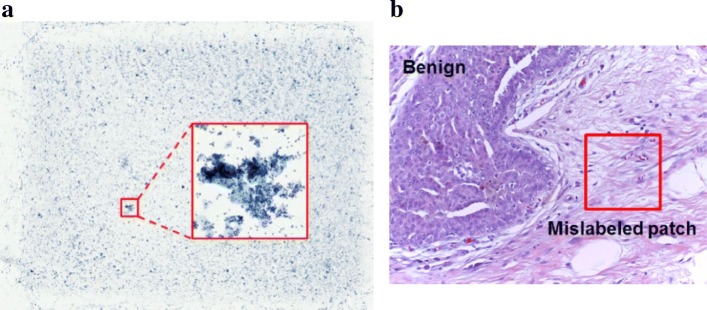
Challenges for pathological image classification. **a** Gigapixel TCT image for cervical carcinoma. **b** An example of a mislabeled patch from the BACH dataset. The normal patch is labeled as benign

First, most pathological images have high resolutions (gigapixels). Figure 1a shows an example of a ThinPrep Cytology Test (TCT) image for cervical carcinoma. The resolution of the TCT image is 21,163×16,473, which is difficult for the CNN to process directly. Second, the number of pathological images contained in publicly available datasets are often very limited. For example, the dataset used in the 2018 grand challenge on breast cancer histology images (BACH) consists of 400 images in four categories, with only 100 images available in each category. Hence, the number of training images may not be sufficient to train a deep learning network. Third, most of the pathological images only have the slice-level labels. To address the first two problems, researchers usually crop patches from the whole-slice pathological images to simultaneously decrease the training image size and increase their number. As only the slice-level label is available, the label pertaining to the whole-slice is usually assigned to the associated patches. However, tumors may have a mix of structure and texture properties [3], and there may be normal tissues around tumors. Hence, the patch-level labels may be inconsistent with the slice-level label. Figure 1b shows an example of a breast cancer histology image. The slice label is assigned to the normal patch marked with red square. Such mislabeled patches may influence the subsequent network training and decrease classification accuracy.

In this paper, we propose a deep learning framework to classify the pathological images. The main contributions can be summarized as follows:

1) An active learning strategy is proposed to remove mislabeled patches from the training set for deep learning networks. Compared to the typical active learning that iteratively trains a model with the incrementally labeled data, the proposed strategy - deep-reverse active learning (DRAL) - can be seen as a reverse of the typical process.

2) An advanced network architecture - atrous DenseNet (ADN) - is proposed for classification of the pathological images. We replace the common convolution of DenseNet with the atrous convolution to achieve multiscale feature extraction.

3) Experiments are conducted on three pathological datasets. The results demonstrate the outstanding classification accuracy of the proposed DRAL + ADN framework.

### Active Learning

Active learning (AL) aims to decrease the cost of expert labeling without compromising classification performance [4]. This approach first selects the most ambiguous/uncertain samples in the unlabeled pool for annotation and then retrains the machine learning model with the newly labeled data. Consequently, this augmentation increases the size of the training dataset. Wang [4] proposed the first active learning approach for deep learning. The approach used three metrics for data selection: least confidence, margin sampling, and entropy. Rahhal et al. [5] suggested using entropy and Breaking-Ties (BT) as confidence metrics for selection of electrocardiogram signals in the active learning process. Researchers recently began to employ active learning for medical image analysis. Yang [6] proposed an active learning-based framework - a stack of fully convolutional networks (FCNs) - to address the task of segmentation of biomedical images. The framework adopted the FCNs results as the metric for uncertainty and similarity. Zhou [7] proposed a method called active incremental fine-tuning (AIFT) to integrate active learning and transfer learning into a single framework. The AIFT was tested on three medical image datasets and achieved satisfactory results. Nan [8] made the first attempt at employing active learning for analysis of pathological images. In this study, an improved active learning based framework (reiterative learning) was proposed to leverage the requirement of a human prediction.

Although active learning is an extensively studied area, it is not appropriate for the task of patch-level pathological image classification. The aim of data selection for patch-level pathological image classification is to remove the mislabeled patches from the training set, which is different from the traditional active learning, i.e., incremental augmentation of the training set. To address this challenge, we propose deep-reverse active learning (DRAL) for patch-level data selection. We acknowledge that the idea of reverse active learning has been proposed in 2012 [9]. Therefore, we hope to highlight the difference between the RAL proposed in that study and ours. First, the typical RAL [9] is proposed for clinical language processing, while ours is for 2-D pathological images. Consequently, the criteria for removing mislabeled (negative) samples are totally different. Second, the typical RAL [9] is developed on the LIBSVM software. In contrast, we adopt the deep learning network as the backbone of the machine learning algorithm, and remove the noisy samples by using the data augmentation approach of deep learning.

### Deep Learning-based Pathological Image Analysis

The development of the deep convolutional network was inspired by Krizhevsky, who won the ILSVRC 2012 competition with the eight-layer AlexNet [1]. In the following competitions, a number of new networks such as VGG [10] and GoogLeNet [11], were proposed. He et al. [12], the ILSVRC 2015 winner, proposed a much deeper convolutional network, ResNet, to address the training problem of ultradeep convolutional networks. Recently, the densely connected network (DenseNet) proposed by Huang [13] outperformed the ResNet on various datasets.

In recent years, an increasing number of deep learning-based computer-aided diagnosis (CAD) models for pathological images have been proposed. Albarqouni [14] developed a new deep learning network, AggNet, for mitosis detection in breast cancer histology images. A completely data-driven model that integrated numerous biological salient classifiers was proposed by Shah [15] for invasive breast cancer prognosis. Chen [16] proposed a framework based on FCN for segmentation of glands. Li [17] proposed an ultradeep residual network for segmentation and classification of human epithelial type-2 (HEp-2) specimen images. More recently, Liu [18] developed an end-to-end deep learning system to directly predict the H-Score for breast cancer tissue. All the aforementioned algorithms crop patches from pathological images to augment the training set, and achieve satisfactory performance on specific tasks. However, we noticed that few of the presented CAD systems use the DenseNet state-of-the-art network architecture, which leaves some margin for performance improvement. In this paper, we propose a deep neural network called ADN for analysis of pathological images. The proposed framework significantly outperforms the benchmark models and achieves excellent classification accuracy on two types of pathological datasets: breast and cervical slices.

### Atrous Convolution & DenseNet

The proposed atrous DenseNet (ADN) is inspired by atrous convolution (or dilated convolution) and the DenseNet state-of-the-art network architecture [13]. In this section, we first present the definitions of atrous convolution and the original dense block.

#### Atrous Convolution

**Fig. 2:**
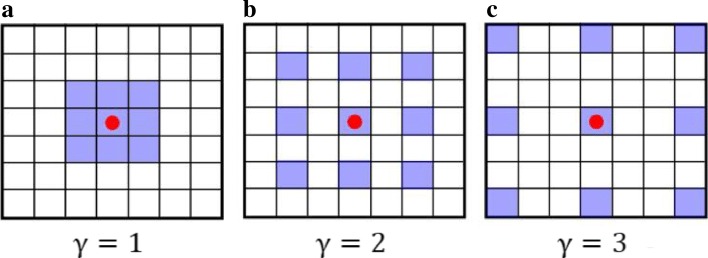
Examples of atrous convolutions with different dilation rates. The purple squares represent the positions of kernel parameters

The atrous convolution (or dilated convolution) was employed to improve the semantic segmentation performance of deep learning based models [19]. Compared to the common convolution layer, the convolutional kernels in the atrous convolution layer have “holes” between parameters that enlarge the receptive field without increasing the number of parameters. The size of the “holes” inserted into the parameters is calculated based on the dilation rate (*γ*). As shown in Fig. 2, a smaller dilation rate results in a more compact kernel (the common convolution can be seen as a special case with dilation rate = 1), while a larger dilation rate produces an expanded kernel. A kernel with a larger dilation rate can capture more context information from the feature maps of the previous layer.

#### Dense Block

The dense block adopted in the original DenseNet is introduced in [13]. Let *H*_*l*_(.) be a composite function of operations such as convolution and rectified linear units (ReLU), the output of the *l*^*th*^ layer (*x*_*l*_) for a single image *x*_0_ can be written as follows:

1 $$\begin{array}{@{}rcl@{}} x_{l} = H_{l}([x_{0},x_{1},...,x_{l-1}]) \end{array} $$

where [*x*_0_,*x*_1_,...,*x*_*l*−1_] refers to the concatenation of the feature maps produced by layers 0,...,*l*−1.

If each function *H*_*l*_(.) produces k feature maps, the *l*^*th*^ layer consequently has *k*_0_+*k*×(*l*−1) input feature maps, where *k*_0_ is the number of channels of the input layer. *k* is called growth rate of the DenseNet block.

## Methods

### Deep-Reverse Active Learning

To detect and remove the mislabeled patches, we propose a reversed process of traditional active learning. As overfitting of deep networks may easily occur, a simple six-layer CNN called RefineNet (RN) is adopted for our DRAL (see the appendix for the architecture). Let M represent the RN model in the CAD system, and let D represent the training set with m patches (x). The deep-reverse active learning (DRAL) process is illustrated in Algorithm 1.


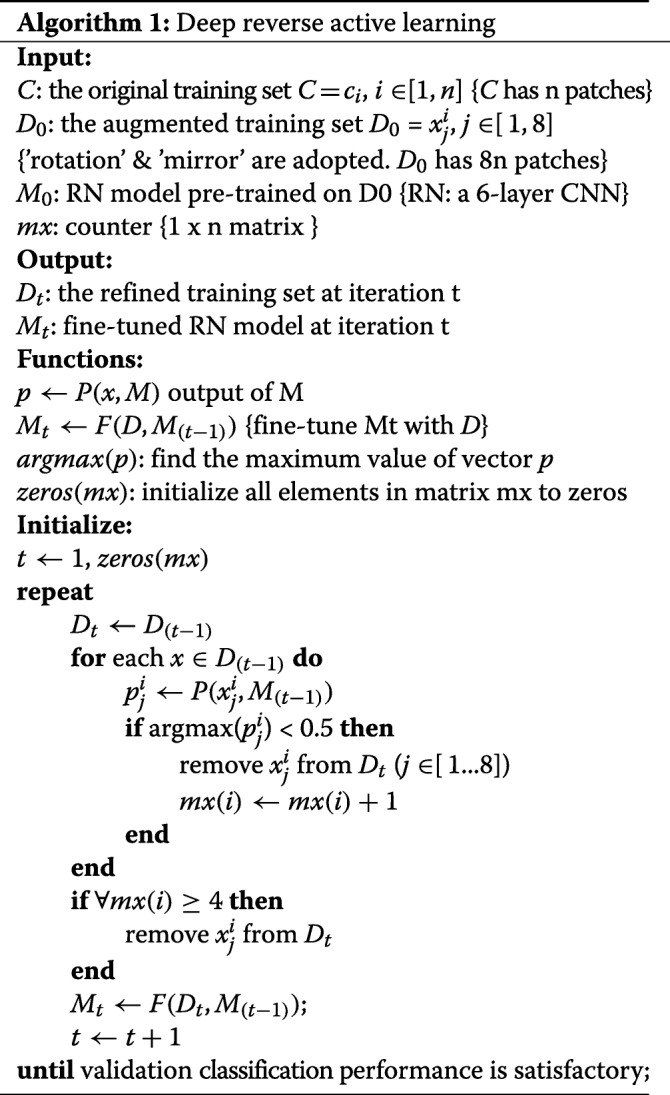


The RN model is first trained, and then makes predictions on the original patch-level training set. The patches with maximum confidence level lower than 0.5 are removed from the training set. As each patch is augmented to eight patches using data augmentation (“rotation” and “mirror”), if more than four of the augmented patches are removed, then the remaining patches are removed from the training set. The patch removal and model fine-tuning are performed in alternating sequence. A fixed validation set annotated by pathologists is used to evaluate the performance of fine-tuned model. Using DRAL resulted in a decline in the number of mislabeled patches. As a result, the performance of the RN model on the validation set is gradually improved. The DRAL stops when the validation classification accuracy is satisfactory or stops increasing. The training set filtered by DRAL can be seen as correctly annotated data, and can be used to train deeper networks such as ResNet, DenseNet, etc.

### Atrous DenseNet (ADN)

**Fig. 3:**
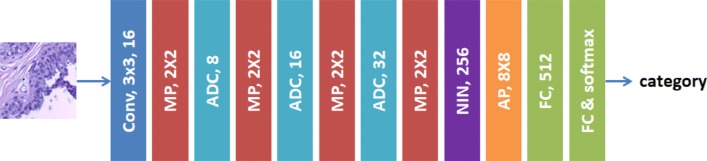
Network architecture of the proposed atrous DenseNet (ADN). Two modules (atrous dense connection (ADC) and network-in-network (NIN)) are involved in the ADN. The blue, red, orange and green rectangles represent the convolution, max pooling, average pooling and fully connected layers, respectively

The size of cancer areas in pathological images varies widely. To better extract multiscale features, we propose a deep learning architecture - atrous DenseNet - for pathological image classification. Compared to common convolution kernels [11], atrous convolutions can extract multiscale features without extra computational cost. The network architecture is presented in Fig. 3.

The blue, red, orange and green rectangles represent the convolutional layer, max pooling layer, average pooling layer and fully connected layers, respectively. The proposed deep learning network has different architectures for shallow layers (atrous dense connection (ADC)) and deep layers (network-in-network module (NIN) [20]). PReLU is used as the nonlinear activation function. The network training is supervised by the softmax loss (L), as defined in Eq. 2 as follows:

2 $$\begin{array}{@{}rcl@{}} L = \frac{1}{N} \sum_{i} L_{i} = \frac{1}{N} \sum_{i} - log(\frac{e^{f_{y_{i}}}}{\sum_{j}e^{f_{j}}}) \end{array} $$

where *f*_*j*_ denotes the *j*^*th*^ element (*j*∈[1,*K*], *K* is the number of classes) of vector of class scores f, *y*_*i*_ is the label of *i*^*th*^ input feature and *N* is the number of training data.

Our ADC proposes to use atrous convolution to replace the common convolution in the original DenseNet blocks and a wider DenseNet architecture is designed by using wider densely connected layers.

#### Atrous Convolution Replacement

**Fig. 4:**
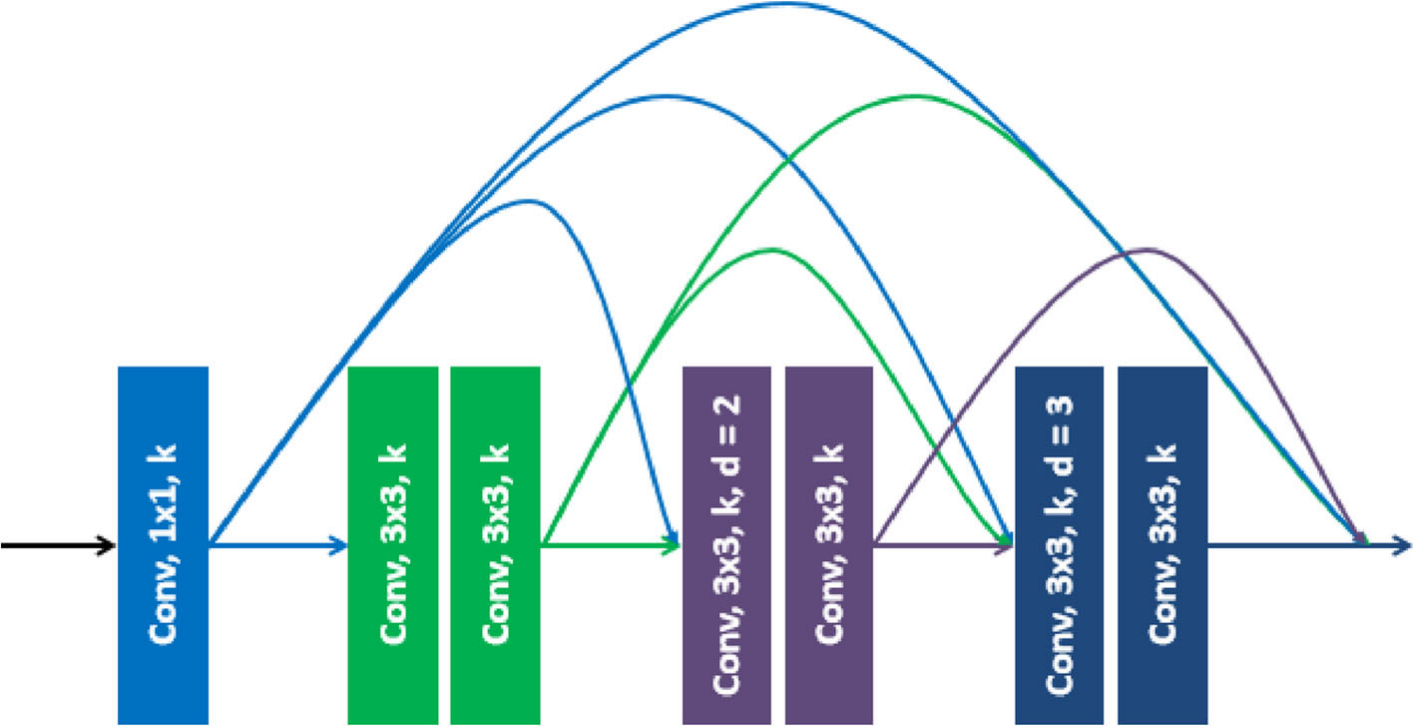
Network architecture of the proposed atrous dense connection (ADC). Convolutions with different dilation rates are adopted for multiscale feature extraction. The color connections refer to the feature maps produced by the corresponding convolution layers. The feature maps from different convolution layers are concatenated to form a multiscale feature

The original dense block achieved multiscale feature extraction by stacking 3×3 convolutions. As the atrous convolution has a larger receptive field, the proposed atrous dense connection block replaces the common convolutions with the atrous convolution to extract better multiscale features. As shown in Fig. 4, atrous convolutions with two dilation rates (2 and 3) are involved in the proposed ADC block. The common 3×3 convolution is placed after each atrous convolution to fuse the extracted feature maps and refine the semantic information.

We notice that some studies have already used the stacking atrous convolutions for semantic segmentation [21]. The proposed ADC addresses two primary drawbacks of the existing framework. First, the dilation rates used in the existing framework are much larger (2, 4, 8 and 16) compared to the proposed ADC block. As a result, the receptive field of the existing network normally exceeds the patch size and requires multiple zeros as padding for the convolution computation. Second, the architecture of the existing framework has no shortcut connections, which is not appropriate for multiscale feature extraction.

#### Wider Densely Connected Layer

As the numbers of pathological images in common datasets are usually small, it is difficult to use them to train an ultradeep network such as the original DenseNet. Zagoruyko [22] proved that a wider network may provide better performance than a deeper network when using small datasets. Hence, the proposed ADC increases the growth rate (k) from 4 to 8, 16 and 32, and decreases the number of layers (l) from 121 to 28. Thus, the proposed dense block is wide and shallow. To reduce the computational complexity and enhance the capacity of feature representation, the growth rate (the numbers in the ADC modules in Fig. 3) increases as the network goes deeper.

### Implementation

To implement the proposed ADN, the Keras toolbox is used. The network was trained with a mini-batch of 16 on four GPUs (GeForce GTX TITAN X, 12GB RAM). Due to the use of batch normalization layers, the initial learning rate was set to a large value (0.05) for faster network convergence. Following that, the learning rate was decreased to 0.01, and then further decreased with a rate of 0.1. The label for a whole-slice pathological image (slice-level prediction) is rendered by fusing the patch-level predictions made by ADN (voting).

## Results

### Datasets

Three datasets are used to evaluate the performance of the proposed model: the BreAst Cancer Histology (BACH), Cervical Carcinoma Grade (CCG), and UCSB breast cancer datasets. While independent test sets are available for BACH and CCG, only a training and validation set are available for UCSB due to the limited number of images. While training and validation sets for the three datasets are first used to evaluate the performance of the proposed DRAL and ADN against popular networks such as AlexNet, VGG, ResNet and DenseNet, the independent test sets are used to evaluate the performance of the proposed approach against the state-of-the-art approach using public testing protocols.

#### BreAst Cancer Histology dataset (BACH)

**Fig. 5:**
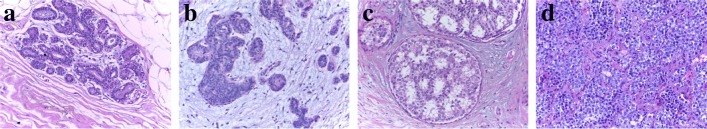
Examples from the BreAst Cancer Histology dataset (BACH). **a** Normal slice, **b** Benign slice, **c** Carcinoma in situ, **d** Invasive carcinoma slice

The BACH dataset [23] consists of 400 pieces of 2048×1536 Hematoxylin and Eosin (H&E) stained breast histology microscopy images, which can be divided into four categories: normal (Nor.), benign (Ben.), in situ carcinoma (C. in situ), and invasive carcinoma (I. car.). Each category has 100 images. The dataset is randomly divided with an 80:20 ratio for training and validation. Examples of slices from the different categories are shown in Fig. 5. The extra 20 H&E stained breast histological images from the Bioimaging dataset [24] are adopted as a testing set for the performance comparison of our framework and benchmarking algorithms.

We slide the window with a 50% overlap over the whole image to crop patches with a size of 512×512. The cropping produces 2800 patches for each category. Rotation and mirror are used to increase the training set size. Each patch is rotated by 90^∘^, 180^∘^ and 270^∘^ and then reflected vertically, resulting in an augmented training set with 896,000 images. The slice-level labels are assigned to the generated patches.

#### Cervical Carcinoma Grade dataset (CCG)

**Fig. 6:**
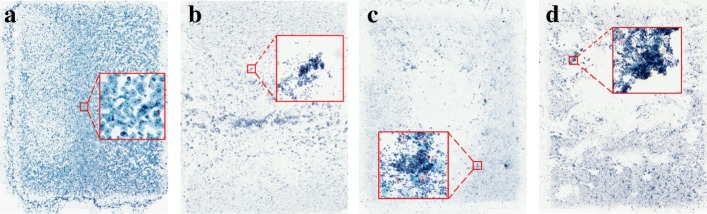
Examples from the Cervical Carcinoma Grade dataset (CCG). **a** Normal slice, **b** Cancer-level I slice, **c** Cancer-level II slice, **d** Cancer-level III slice. The resolution of the slices is in gigapixels, i.e., 16,473×21,163. The areas in red squares have been enlarged for illustration

The CCG dataset contains 20 H&E-stained whole-slice ThinPrep Cytology Test (TCT) images, which can be classified in four grades: normal and cancer-level I (L. I), II (L. II), III (L. III). The five slices in each category are separated according to a 60:20:20 ration for training, validation and testing. The resolution of the TCT slices is 16,473×21,163. Figure 6 presents a few examples of slices from the different categories. The CCG dataset is populated by pathologists collaborating on this project using a whole-slice scanning machine.

We crop the patches from the gigapixel TCT images to generate the patch-level training set. For each normal slice, approximately 20,000 224×224 patches are randomly cropped. For the cancer slices (Fig. 6b-d), as they have large background areas, we first binarize the TCT slices to detect the region of interest (RoI). Then, the cropping window is passed over the RoI for patch generation. The slice-level label is assigned to the produced patches. Rotation is used to increase the size of training dataset. Each patch is rotated by 90^∘^, 180^∘^ and 270^∘^ to generate an augmented training set with 362,832 images. The patch-level validation set consists of 19,859 patches cropped from the validation slices. All of them have been verified by the pathologists. The detailed information of patch-level CCG dataset is presented in Table 1.

**Table 1:** Detailed information of CCG dataset

	

#### UCSB Breast Cancer dataset

**Fig. 7:**
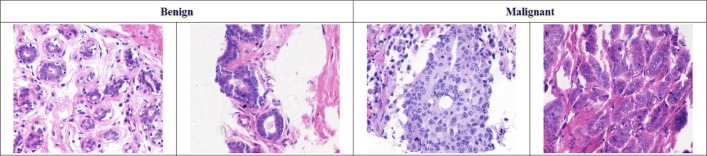
Examples from the UCSB dataset. The dataset has 32 benign slices and 26 malignant slices

The UCSB dataset contains 58 pieces of 896×768 breast cancer slices, which can be classified as benign (Ben.) (32) or malignant (Mal.) (26). The dataset is divided into training and validation sets according to a 75:25 ratio. Examples of UCSB images are shown in Fig. 7. We slide a 112×112 window over the UCSB slices to crop patches for network training and employ the same approach used for BACH to perform data augmentation. As many studies have reported their 4-fold cross validation results on UCSB dataset, we also conduct the same experiment for fair comparison.

#### Discussion of Preprocessing Approaches for Different Datasets

As previously mentioned, the settings for the preprocessing approaches (including the size of cropped patches and data augmentation) are different for each dataset. The reason is that the image size and quantity in each dataset are totally different. To generate more training patches, we select a smaller patch size (112×112) for the dataset with fewer lower resolution samples (UCSB) and a larger one (512×512) for the dataset with high-resolution images (BACH). For the data augmentation, we use the same data augmentation approach for the BACH and UCSB datasets. For the CCG dataset, the gigapixel TCT slices can yield more patches than the other two datasets. While horizontal and vertical flipping produce limited improvements in classification accuracy, they significantly increase the time cost of the network training. Hence, we only adopt three rotations to augment the training patches of the CCG dataset.

### Evaluation Criterion

The overall correct classification rate (ACA) of all the testing images is adopted as the criterion for performance evaluation. In this section, we will first evaluate the performance of DRAL and ADN on the BACH, CCG, and UCSB validation sets. Next, the results from applying different frameworks to the separate testing sets will be presented. Note that the training and testing of the neural networks are performed three times in this study, and the average ACAs are reported as the results.

### Evaluation of DRAL

#### Classification Accuracy during DRAL

**Fig. 8:**
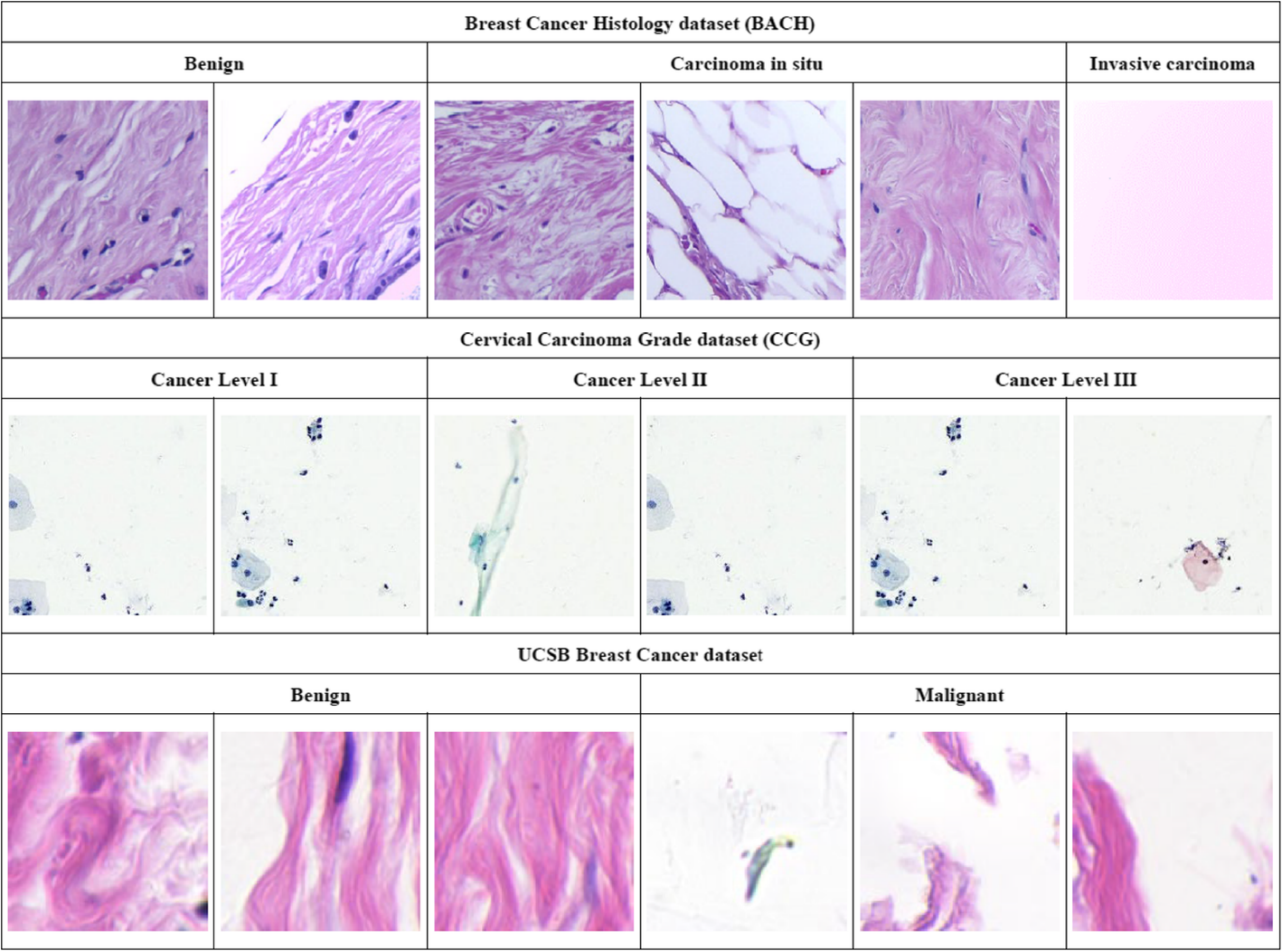
Illustrations of mislabeled patches. The first, second and third rows list the normal patches mislabeled as cancer from the BACH, CCG, and UCSB datasets, respectively. All the patches have been verified by pathologists

The proposed DRAL adopts RefineNet (RN) to remove mislabeled patches from the training set. As presented in Table 2, the size of training set decreases from 89,600 to 86,858 for BACH, from 362,832 to 360,563 for CCG, and from 68,640 to 64,200 for UCSB. Figure 8 shows some examples of mislabeled patches identified by the DRAL; most of them are normal patches labeled as breast or cervical cancer. The ACAs on the validation set during the patch filtering process are presented in Table 2. It can be observed that the proposed DRAL significantly increases the patch-level ACAs of RN: the improvements for BACH, CCG, and UCSB are 3.65%, 6.01%, and 17.84%, respectively.

**Table 2:** Patch-level ACA (P. ACA, %) of RN on Validation Sets during Different Iterations of DRAL

DRAL (Iteration number K)	BACH		CCG		UCSB	
	Training set	P. ACA	Training set	P. ACA	Training set	P. ACA
trained with original training set (K=0)	89,600	89.16	362,832	77.87	68,640	76.40
K=1	89,026	89.58	361,007	83.88	64,944	94.24
K=2	88,170	89.71	360,563	82.88	64,200	93.23
K=3	87,363	92.81	-	-	-	-
K=4	86,858	92.14	-	-	-	-

**Fig. 9:**
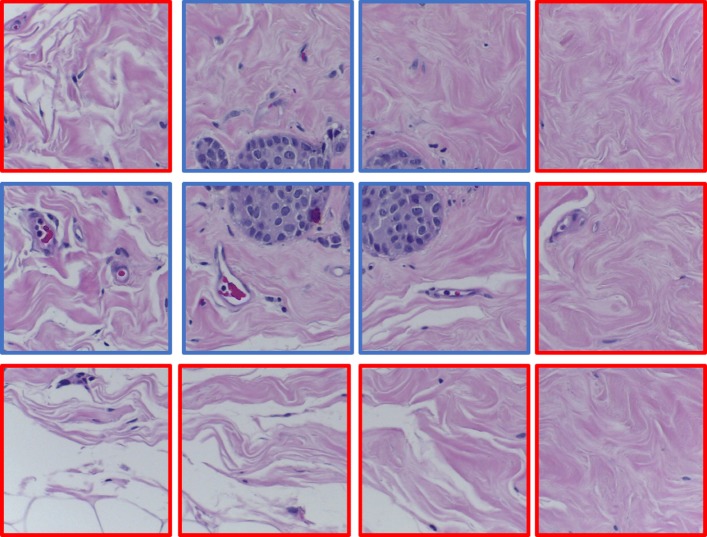
Examples of retained and discarded patches of BACH images. The patches marked with red and blue boxes are respectively recognized as “mislabeled” and “correctly annotated” by our RAL

To better analyze the difference between the patches retained and discarded by our DRAL, an example of a BACH image containing the retained and discarded patches is shown in Fig. 9. The patches with blue and red boxes are respectively marked as “correctly annotated” and “mislabeled” by our DRAL. It can be observed that patches in blue boxes contain parts of breast tumors, while those in the red boxes only contain normal tissues.

**Fig. 10:**
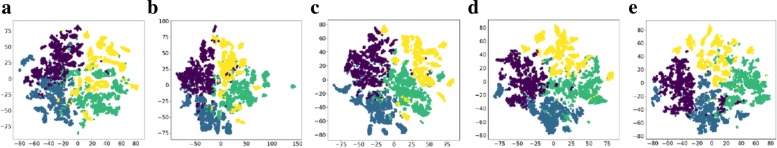
The t-SNE figures of the last fully connected layer of RefineNet for different iterations K of the BACH training process. **a**-**e** are for K = 0, 1, 2, 3, 4, respectively

In Fig. 10, the t-SNE [25] is used to evaluate the RefineNet’s capacity for feature representation during different iterations of the BACH training process. The points in purple, blue, green and yellow respectively represent the normal, benign, carcinoma in situ, and invasive carcinoma samples. It can be observed that the RefineNet’s capacity for feature representation gradually improved (the different categories of samples are gradually separated during DRAL training). However, Fig. 10e shows that the RefineNet, after the fourth training iteration (K=4), leads to the misclassification of some carcinoma in situ (green) and normal samples (purple) as invasive carcinoma (yellow) and carcinoma in situ (green), respectively.

#### CNN Models trained with the Refined Dataset

The DRAL refines the training set by removing the mislabeled patches. Hence, the information contained in the refined training set is more accurate and discriminative, which is beneficial for the training of a CNN with deeper architecture. To demonstrate the advantages of the proposed DRAL, several well-known deep learning networks such as AlexNet [1], VGG-16 [10], ResNet-50/101 [12], and DenseNet-121 [13] are used for the performance evaluation. These networks are trained on the original and refined training sets and also evaluated on the same fully annotated validation set. The evaluation results are presented in Table 3 (Patch-level ACA) and Table 4 (Slice-level ACA).

**Table 3:** Patch-level Validation ACA (%) of CNN Models Trained on The Original/Refined Training Sets

	BACH	CCG		UCSB		
	Original	Refined	Original	Refined	Original	Refined
AlexNet [1]	86.28	90.77	75.67	82.24	76.51	93.78
VGG-16 [10]	90.83	91.79	84.63	90.02	83.53	97.44
ResNet-50 [12]	89.65	92.17	79.88	82.31	78.74	96.82
ResNet-101 [12]	89.05	91.17	80.06	83.47	77.82	96.78
DenseNet [13]	90.39	93.29	77.87	84.41	78.93	96.79
ADN (ours)	**91.93**	**94.10**	**85.48**	**92.05**	**85.69**	**97.63**

Best accuracy is in Bold.

**Table 4:** Slice-level Validation ACA (%) of CNN Models Trained on The Original/Refined Training Sets

	BACH		CCG		UCSB	
	original	refined	original	refined	original	refined
AlexNet [1]	86.25	91.25	50	75	80	90
VGG-16 [10]	87.50	96.25	**75**	75	**90**	**100**
ResNet-50 [12]	86.25	93.75	**75**	75	80	**100**
ResNet-101 [12]	86.25	91.25	**75**	75	80	90
DenseNet [13]	86.25	96.25	50	75	80	90
ADN (ours)	**88.75**	**97.50**	**75**	**100**	**90**	**100**

Best accuracy is in Bold.

As shown in Tables 3 and 4, for all three datasets, the classification accuracy of networks trained on the refined training set are better than those trained on the original training set. The greatest improvements for the patch-level ACA that used DRAL is 4.49% for AlexNet on BACH, 6.57% for both AlexNet and our ADN on CCG, and 18.91% for VGG on UCSB. For the slice-level ACA, the proposed DRAL improves the performance of our ADN from 88.57% to 97.50% on BACH, from 75% to 100% on CCG, and from 90% to 100% on UCSB.

The results show that mislabeled patches in the original training sets have negative influences on the training of deep learning networks and decrease classification accuracy. Furthermore, the refined training set produced by the proposed DRAL is useful for general, deep learning networks such as shallow networks (AlexNet), wide networks (VGG-16), multibranch deep networks (ResNet-50) and ultradeep networks (ResNet-101 and DenseNet-121).

### Evaluation of Atrous DenseNet (ADN)

Tables 3 and 4 show that our ADN outperforms all the listed networks on BACH, CCG, and UCSB with and without the DRAL. This section presents a more comprehensive performance analysis of the proposed ADN.

#### ACA on the BACH Dataset

**Fig. 11:**
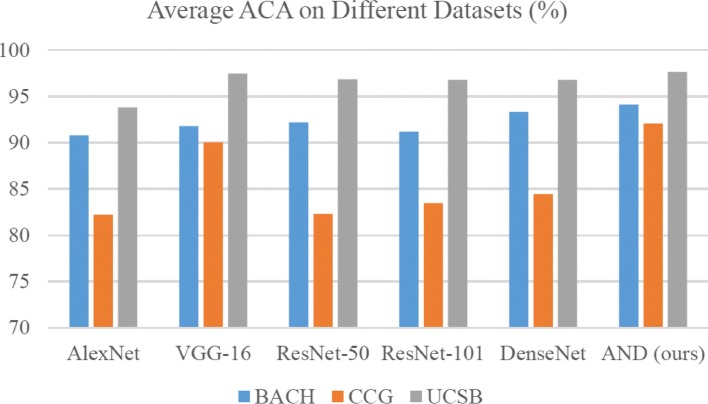
Patch-level average ACA of different deep learning models on three datasets

The patch-level ACA of different CNN models for each category of BACH is listed in Table 5. All the models are trained with the training set refined by DRAL. The average ACA (Ave. ACA) is the overall classification accuracy of the patch-level validation set. The Ave. ACA results are shown in Fig. 11.

**Table 5:** Patch-level ACA (%) for Different Categories of Different Datasets

	BACH				CCG				UCSB	
	Nor.	Ben.	C. in situ	I. car.	Nor.	L. I	L. II	L. III	Ben.	Mal.
AlexNet [1]	92.13	90.18	89.52	91.25	95.16	93.68	95.82	42.43	94.81	92.75
VGG-16 [10]	90.96	**93.84**	89.46	92.89	98.71	96.36	98.06	65.61	98.32	96.58
ResNet-50 [12]	92.29	94.50	92.29	91.61	87.54	**99.10**	92.87	50.32	97.48	96.16
ResNet-101 [12]	91.96	89.20	90.66	92.88	85.46	98.32	**99.88**	50.45	98.07	95.49
DenseNet [13]	94.61	91.50	**95.73**	93.82	92.04	98.05	96.97	50.08	96.97	96.60
ADN (ours)	**96.30**	92.36	93.50	**94.23**	**99.18**	97.70	99.52	**70.68**	**98.54**	**96.73**

Best accuracy is in Bold.

As shown in Table 5, the proposed ADN achieves the best classification accuracy for the normal (96.30%) and invasive carcinoma (94.23%) patches, while the ResNet-50 and DenseNet-121 yield the highest ACAs for benign (94.50%) and carcinoma in situ (95.73%) patches. The ACAs of our ADN for benign and carcinoma in situ are 92.36% and 93.50%, respectively, which are competitive compared to the performance of other state-of-the-art approaches. The average ACA of ADN is 94.10%, which outperforms the listed benchmarking networks.

**Fig. 12:**
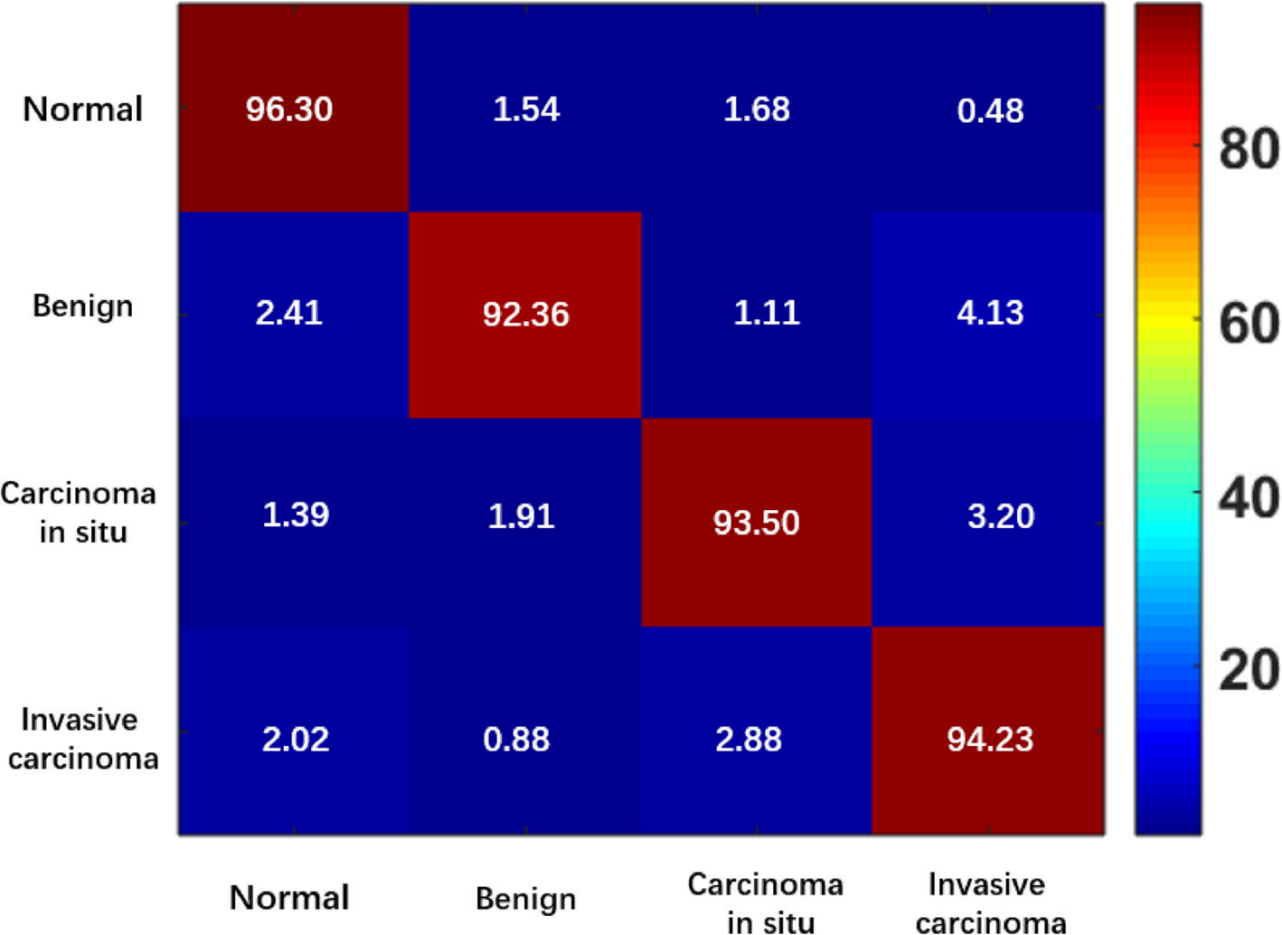
Confusion map of ADN on BACH validation set

To further evaluate the performance of the proposed ADN, its corresponding confusion map on the BACH validation set is presented in Fig. 12, which illustrates the excellent performance of the proposed ADN for classifying breast cancer patches.

#### ACA on the CCG Dataset

The performance evaluation is also conducted on CCG validation set, and Table 5 presents the experiment results. For the patches cropped from normal and level III slices, the proposed ADN achieves the best classification accuracy (99.18% and 70.68%, respectively), which are 0.47% and 2.03% higher than the runner-up (VGG-16). The best ACAs for level I and II patches are achieved by ResNet-50 (99.10%) and ResNet-101 (99.88%), respectively. The proposed ADN generates competitive results (97.70% and 99.52%) for these two categories.

**Fig. 13:**
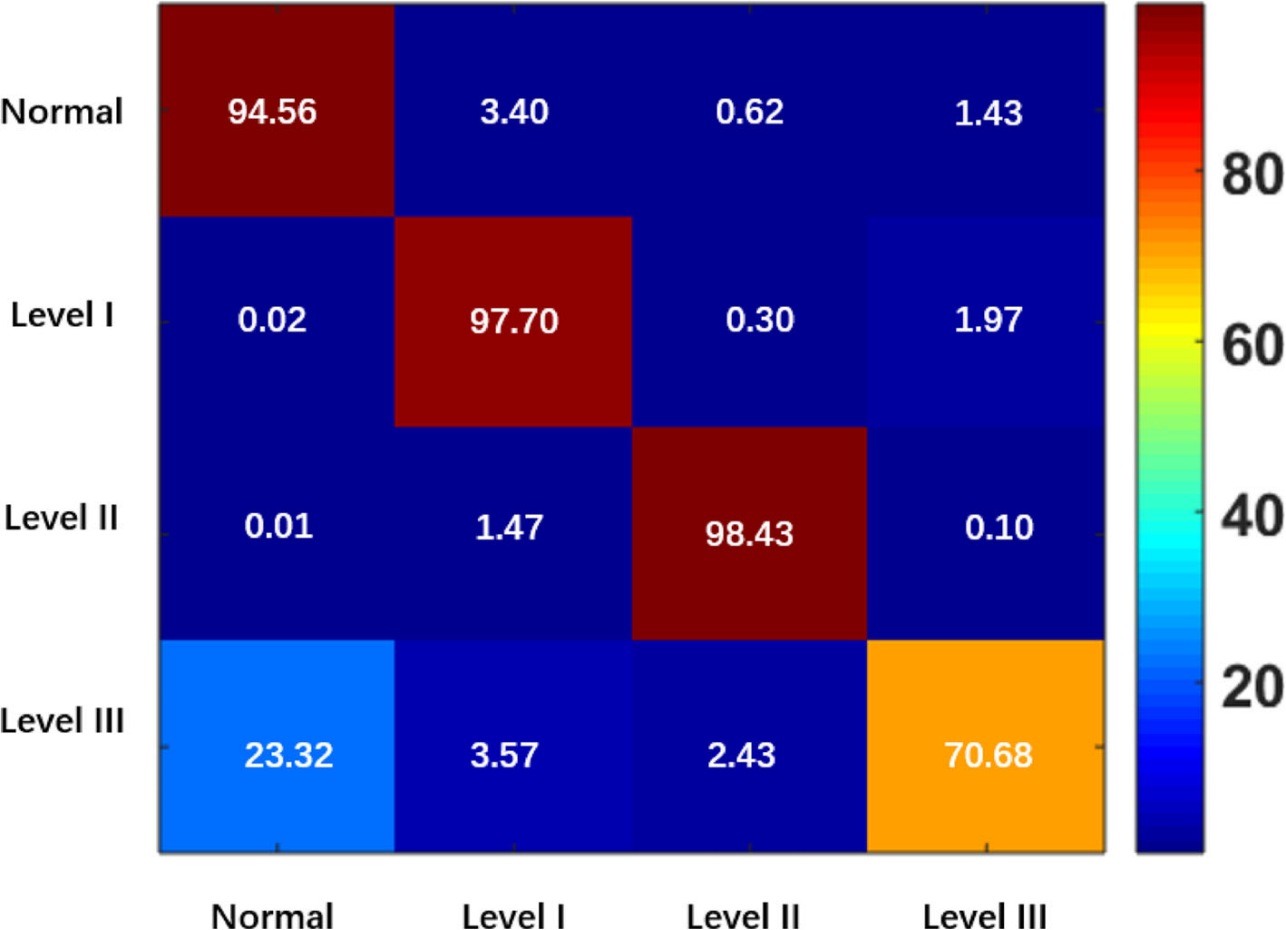
Confusion map of ADN on CCG validation set

All the listed algorithms have low levels of accuracy for the patches from level III slices. To analyze the reasons for this low accuracy, the confusion map for the proposed ADN is presented in Fig. 13. It can be observed that some cancer level III patches are incorrectly classified as normal. A possible reason is that the tumor area in cancer level III is smaller than that of cancer levels I and II, so patches cropped from cancer level III slices usually contain normal areas. Therefore, the level III patches with large normal areas may be recognized as normal patches by ADN. We evaluated the other deep learning networks and again found that they incorrectly classify the level III patches as normal. To address the problem, a suitable approach that fuses the patch-level predictions with slice-level decisions needs to be developed.

#### ACA on the UCSB Dataset

Table 5 lists the patch-level ACAs of different deep learning frameworks on the UCSB validation set. It can be observed that our ADN achieves the best patch-level ACAs; 98.54% (benign) and 96.73% (malignant). The runner-up (VGG-16) achieves patch-level ACAs of 98.32% and 96.58%, which are 0.22% and 0.15% lower than the proposed ADN. The ResNet-50/101 and DenseNet yield similar performances (average ACAs are approximately 96%), while the AlexNet generates the lowest average ACA of 93.78%.

#### Statistical Validation

A T-test validation was conducted for the results from VGG-16 and our ADN. The p-values at the 5% significance level are 1.07%, 2.52% and 13.08% for BACH, CCG, and UCSB, respectively. The results indicate that the accuracy improvement is statistically significant for BACH and CCG. As the number of images (58) in UCSB is quite small, the problem might not be challenging enough. Therefore, both VGG-16 and our ADN achieve similar performances. Consequently, the deep learning networks yield similar classification accuracy levels on the UCSB dataset; that is, no statistical significance is observed between the results produced by different models.

#### Network Size

As previously mentioned, instead of building a deeper network, the proposed ADN adopts wider layers to increase its feature representation capacity, which is more suitable for small datasets. To further illustrate the excellent capacity of the proposed ADN, a comparison of network size between different network architectures is presented in Table 6.

**Table 6:** Detailed Information of Different Network Architectures

	No. of Layers	Model Size
AlexNet [1]	8	54 M
VGG-16 [10]	16	158 M
ResNet-50 [12]	50	270 M
ResNet-101 [12]	101	488 M
DenseNet [13]	121	539 M
ADN (ours)	28	132 M

In the experiments, the wider networks - VGG-16 (16 layers) and ADN (28 layers) - achieved better performances than the ultradeep networks - ResNet-50/101 (50/101 layers) and DenseNet (121 layers). Since the VGG-16 and ADN have a much smaller model size than the ultradeep networks, they require fewer network parameters and have a lower risk of overfitting to a small dataset.

Compared to the straightforward VGG-16, the proposed ADN uses multiple atrous convolutions to extract multiscale features. As shown in Fig. 11, the proposed ADN outperforms the VGG-16 and produces the best average ACAs for the BACH (94.10%), CCG (92.05%) and UCSB (97.63%) datasets. The experiment results also demonstrate that the proposed ADN can maintain the balance between network size and feature learning capacity, which is extremely effective for small pathological datasets.

### Comparison with State-of-the-art approaches

In this section, we compare the performance of the proposed framework with other state-of-the-art approaches on the BACH, CCG, and UCSB testing sets. For the UCSB dataset, the public protocol of 4-fold cross validation is used to make the results directly comparable. For better performance evaluation, we include the F-measure (F-mea.) as an additional evaluation metric for BACH and CCG, which can be defined as:

3 $$\begin{array}{@{}rcl@{}} F-measure = \frac{2 \times Precision \times Recall}{Precision + Recall} \end{array} $$

4 $$\begin{array}{@{}rcl@{}} Precision = \frac{TP}{TP + FP}, \quad Recall = \frac{TP}{TP+FN} \end{array} $$

where *TP*, *FP* and *FN* stand for true positive, false positive and false negative, respectively.

#### Patch-level and Slice-level ACA on BACH

The extra 20 H&E stained breast histological images from a publicly available dataset (Bioimaging [24]) are employed as the testing set for the frameworks trained on BACH. As Bioimaging is a publicly available dataset, the public testing protocol is used and the state-of-the-art results [24] are directly used for comparison. The results on the testing set are listed in Table 7 (Precision (Pre.), Recall (Rec.)).

**Table 7:** ACA (%) of Different Frameworks for BACH Testing Set

	Patch-level					Slice-level			
	Nor.	Ben.	C. in situ	I. car.	Ave. ACA	Ave. ACA	Pre.	Rec.	F-mea.
CNN [24]	61.70	56.70	83.30	88.30	72.50	80	79.52	80.00	79.76
CNN+SVM [24]	65.00	61.70	76.70	88.30	72.93	85	86.61	85.00	85.80
AlexNet [1]	60.00	58.33	85.00	95.00	74.58	80	82.86	80.00	81.40
VGG-16 [10]	**75.00**	61.67	75.00	90.00	75.42	85	86.61	85.00	85.80
ResNet-50 [12]	63.33	65.00	80.00	95.00	75.83	85	86.67	85.00	85.83
ResNet-101 [1]	65.00	70.00	75.00	90.00	75.00	85	87.86	85.00	86.41
DenseNet [13]	66.67	**76.67**	73.33	88.33	76.25	85	90.00	80.33	84.89
ADN (ours)	60.00	66.67	**88.33**	93.33	77.08	85	86.67	85.00	85.83
ADN+DRAL (ours)	71.67	73.33	**88.33**	**96.67**	**82.50**	**90**	**92.86**	**90.00**	**91.41**

Best accuracy is in Bold

As shown in Table 7, the proposed ADN achieves the best average patch-level classification performance (77.08% on the testing set), which is 0.83% higher than the runner-up (DenseNet-121). The ADN trained with the training set refined by DRAL leads to a further improvement of 5.42% for the final classification accuracy. Accordingly, the slice-level average classification accuracy (90%) of the proposed ADN + DRAL framework is the highest among the listed benchmarking algorithms.

#### Patch-level and Slice-level ACA on CCG

The results for the CCG testing set are presented in Table 8. The proposed ADN achieved the best patch-level ACA (80.28%) among the models trained with the original training set, which is 2.51% higher than the runner-up (VGG-16). Furthermore, it has been noticed most of the listed benchmark algorithms do not perform well for the cancer level I patches; the highest accuracy produced by the ultradeep ResNet-101 is only 67.34%. Our ADN achieves a patch-level ACA of 71.51% with a 28-layer architecture.

**Table 8:** ACA (%) of Different Frameworks for CCG Testing Set

	Patch-level					Slice-level	
	Normal	Level I	Level II	Level III	Ave. ACA	Ave. ACA	F-mea.
AlexNet [1]	91.75	42.24	69.88	70.91	68.70	50	41.67
VGG-16 [10]	97.80	63.65	71.25	78.39	77.77	75	66.67
ResNet-50 [12]	97.82	46.86	75.05	68.57	72.08	50	50
ResNet-101 [12]	96.64	67.34	75.57	58.66	74.55	50	41.67
DenseNet [13]	98.81	56.62	72.20	71.04	74.67	75	66.67
ADN (ours)	99.29	71.51	76.51	73.81	80.28	75	66.67
ADN+DRAL (ours)	**99.95**	**80.35**	**85.31**	**82.60**	**87.05**	**100**	**100**

Best accuracy is in Bold

The proposed DRAL refines the training set by removing the mislabeled patches, which benefits the subsequent network training. As a result, the DRAL training strategy yields significant improvements for both average patch-level ACA (6.77%) and average slice-level ACA (25%) when using the proposed ADN framework.

#### Patch-level and Slice-level ACA on UCSB

The 4-fold cross-validation conducted on the UCSB dataset is presented in Table 9. The baselines are obtained using Fisher Vector (FV) descriptors of different local features such as dense SIFT, patchwise DBN, and CNN features from the last convolutional layer (labeled as FV-SIFT, FV-DBN, and FV-CNN). The three FV descriptors are then combined into longer descriptors: S+D (combining FV-SIFT and FV-DBN), S+C (combining FV-SIFT and FV-CNN), D+C (combining FV-DBN and FV-CNN), and S+D+C (combining all three FV descriptors). The linear kernel SVM without dimensionality reduction and the SDR method proposed in [26] are used for classification. Table 9 shows that, our ADN + DRAL achieves the best 4-fold cross-validation accuracy (100%), which outperforms the highest classification accuracy achieved by the benchmark approaches (98.3% yielded by SDR + SVM + FV-CNN).

**Table 9:** 4-Fold Cross Validation (%) of Different Frameworks on UCSB Dataset

	Single FV descriptor			Combination of FV descriptors			
	FV-SIFT	FV-DBN	FV-CNN	S+D	S+C	D+C	S+D+C
SVM	87.9	82.8	96.6	86.2	93.1	91.4	93.1
SDR+SVM [26]	89.7	89.7	98.3	91.4	94.8	96.6	94.8
ADN+DRAL (ours)	**100**						

Best accuracy is in Bold.

## Conclusions

Due to the impressive performance of deep learning networks, researchers find it appealing for application to medical image analysis. However, pathological image analysis based on deep learning networks faces a number of major challenges. For example, most of pathological images have high resolutions - gigapixels. It is difficult for CNN to directly process the gigapixel images, due to the expensive computational costs. Cropping patches from a whole-slice images is the common approach to address this problem. However, most of the pathological datasets only have slice-level labels. While the slice-level labels can be assigned to the cropped patches, the patch-level training sets usually contain mislabeled samples.

To address these challenges, we proposed a framework for pathological image classification. The framework consists of a training strategy - deep-reverse active learning (DRAL) - and an advanced network architecture - atrous DenseNet (ADN). The proposed DRAL can remove the mislabeled patches in the training set. The refined training set can then be used to train widely used deep learning networks such as VGG-16 and the ResNets. A deep learning network - atrous DenseNet (ADN) - is also proposed for the classification of pathological images. The proposed ADN achieves multiscale feature extraction by combining the atrous convolutions and dense blocks.

The proposed DRAL and ADN have been evaluated on three pathological datasets: BACH, CCG, and UCSB. The experiment results demonstrate the excellent performance of the proposed ADN + DRAL framework, achieving average patch-level ACAs of 94.10%, 92.05%, and 97.63% on BACH, CCG, and UCSB validation sets, respectively.

## Appendix A: Architecture of RefineNet

To alleviate the overfitting problem, a simple CNN, namely RefineNet (RN), is adopted in the iterative Reverse Active Learning (RAL) process to remove mislabeled patches. The pipeline of RefineNet is presented in Table 10, which consists of convolutional (C), max pooling (MP), averaging pooling (AP) and fully-connected (FC) layers.

**Table 10:** Architecture of RN

**Layer**	**Type**	**Kernel size & number**
1	C	3×3,16
2	MP	2×2
3	C	3×3,32
4	MP	2×2
5	C	3×3,64
6	MP	2×2
7	C	3×3,64
8	MP	2×2
9	C	3×3,128
10	MP	2×2
11	C	3×3,128
12	AP	7×7
13	FC	256
14	FC	4

Pipeline consists of convolution layer(C), max pooling layer(MP), average pooling layer(AP) and fully-connected layer(FC)

## Data Availability

BACH: https://iciar2018-challenge.grand-challenge.org/ UCSB: https://bioimage.ucsb.edu/research/bio-segmentation

## Abbreviations

ADC: Atrous dense connection
ADN: Atrous DenseNet
Ave. ACA: Average accuracy
BACH: Breast Cancer Histology dataset
Ben.: Benign
C. in situ: in situ carcinoma
CCG: Cervical Carcinoma Grade dataset
DRAL: Deep reversed active learning
F-mea.: F-measure
FV: Fisher vector
I. car.: Invasive carcinoma
L. I: Cancer Level I
L. II: Cancer Level II
L. III: Cancer Level III
Mal.: Malignant
NIN: Network in network module
Nor.: Normal
P. ACA: Patch-level accuracy
Pre.: Precision
Rec.: Recall
RN: RefineNet
TCT: Thinprep cytological test
